# Drug-Coated Balloon Angioplasty for Axillary Artery Stenosis

**DOI:** 10.7759/cureus.22664

**Published:** 2022-02-27

**Authors:** Elias Smirlis, Ala Mustafa, Mostafa Ghanim, Samuel Congello

**Affiliations:** 1 Internal Medicine, MercyOne North Iowa Medical Center, Mason City, USA; 2 Cardiology, MercyOne North Iowa Medical Center, Mason City, USA

**Keywords:** angioplasty, axillary artery, stenosis, balloon, drug-coated balloon

## Abstract

We present a case report of a patient with symptomatic bilateral severe axillary artery stenosis who underwent drug-coated balloon angioplasty. A 59-year-old female with a past medical history of peripheral artery disease presented with bilateral upper extremity claudication on exertion and episodes of syncope. Peripheral angiography showed significant bilateral upper extremity peripheral artery disease (PAD) including bilateral severe axillary artery stenosis. The patient underwent endovascular management with drug-coated balloon angioplasty of the axillary artery bilaterally. Symptoms completely resolved and the patient continues to be on follow-up. Arterial duplex studies on both upper extremities showed no evidence of high-grade stenosis six years after intervention. Drug coated balloon angioplasty can be a successful modality of endovascular management for patients with symptomatic severe axillary artery stenosis. However, more randomized controlled data is required before making any conclusion.

## Introduction

Upper extremity peripheral artery disease (PAD) is common [1]. Although common, PAD of the upper extremities is typically asymptomatic due to abundant collateral circulation [2]. In many cases, the only finding of PAD in the upper extremities is a blood pressure difference of 15 mmHg or more between upper extremities, but more severe cases can present with claudication [2]. Data on endovascular management and outcomes of axillary artery stenosis is limited. This case report presents a patient with symptomatic bilateral severe axillary artery stenosis who underwent drug-coated balloon angioplasty.

## Case presentation

A 59-year-old female has a past medical history of peripheral artery disease, coronary artery disease status post coronary artery bypass graft (CABG), bilateral renal artery stenosis secondary to fibromuscular dysplasia status post bilateral renal artery angioplasty, hypertension, hyperlipidemia, and hypothyroidism. She presented with bilateral upper extremity claudication and episodes of syncope in 2013.

**Figure 1:**
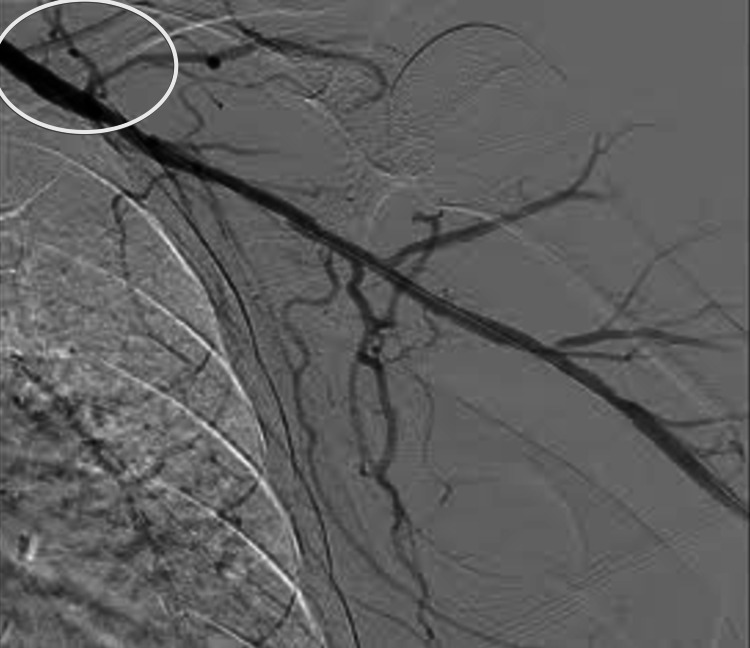
Left axillary artery pre-intervention

**Figure 2:**
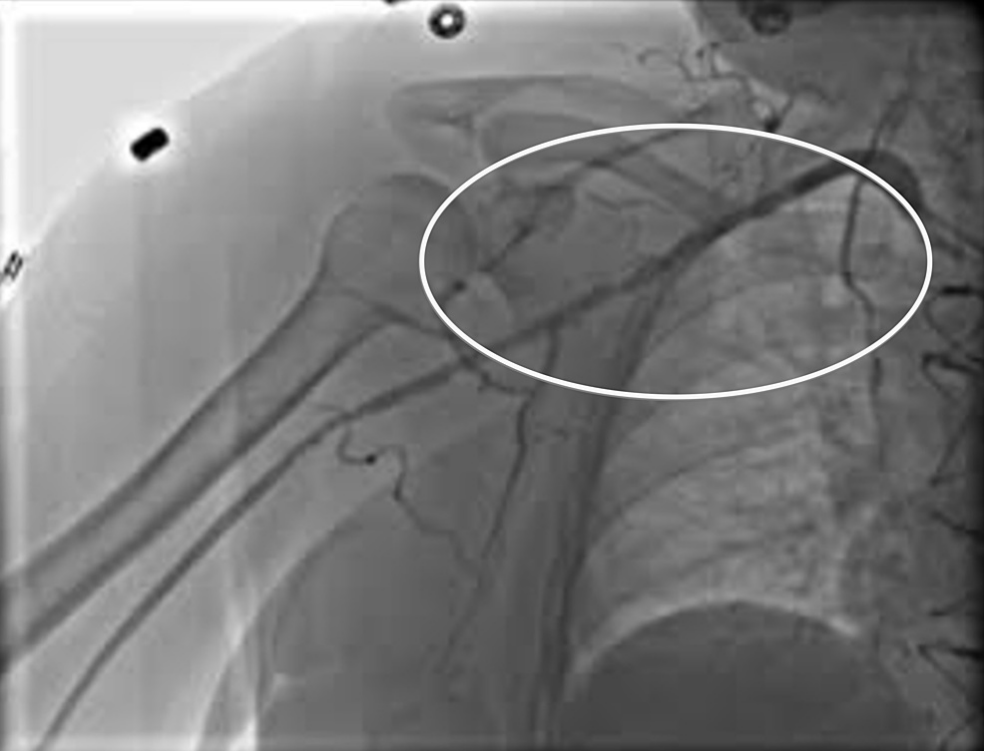
Right axillary artery pre-intervention

The angiography of the upper extremities demonstrated 70% stenosis of the left upper extremity (Figure 1) and multiple areas of stenosis up to 95% in the right upper extremity (Figure 2). Angioplasty of the previously mentioned areas was performed in March 2014. Access was gained through the right femoral artery, and a 4.0 x 100 mm angiosculpt balloon (Spectranetics Corporation, Colorado Springs, USA) was placed and inflated at the left subclavian, axillary, and brachial lesions. The sheath was then redirected to the right subclavian artery ostium, and the angiosculpt balloon was placed and inflated at the right subclavian, axillary, and brachial lesions.

**Figure 3:**
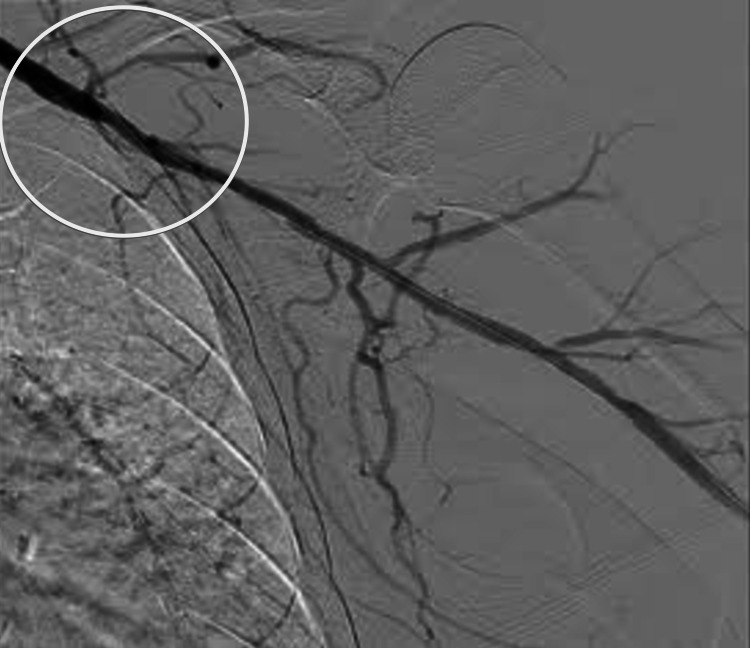
Left axillary artery post-intervention

**Figure 4:**
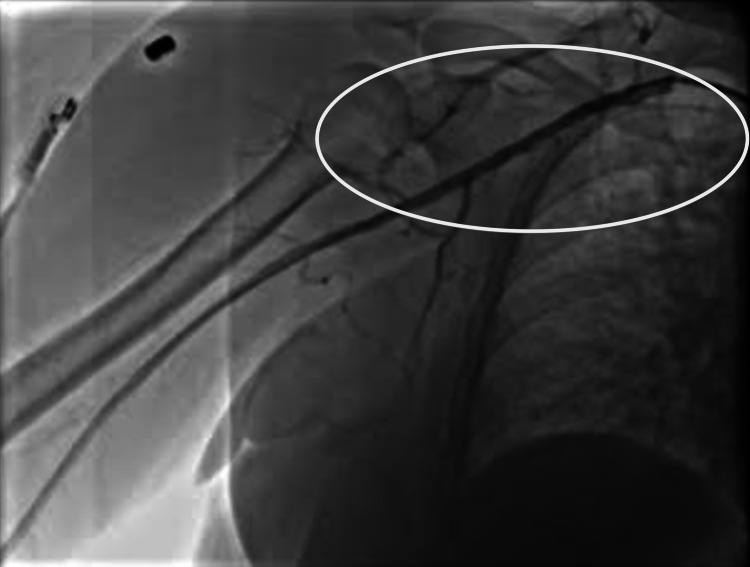
Right axillary artery post-intervention

Post-stenosis results were as follows: left subclavian artery less than 20% occlusion, left axillary artery less than 20% occlusion (Figure 3), left brachial artery less than 20% occlusion, right subclavian artery less than 30% occlusion, right axillary artery less than 20% occlusion (Figure 4), and right brachial artery less than 20% occlusion.

In September 2014, the patient had a repeat noninvasive venous (NV) duplex ultrasound of the upper extremity arteries bilaterally, which revealed elevated velocities and turbulent flow within the left subclavian and axillary arteries. These results led to repeat revascularization in February 2015 with a 4.0 x 100 mm impact drug-eluting balloon (Spectranetics Corporation, Colorado Springs, USA) in the left subclavian and axillary lesions. Repeat noninvasive duplex ultrasound of the upper extremity arteries showed bilateral triphasic waveforms.

Follow-up

**Figure 5:**
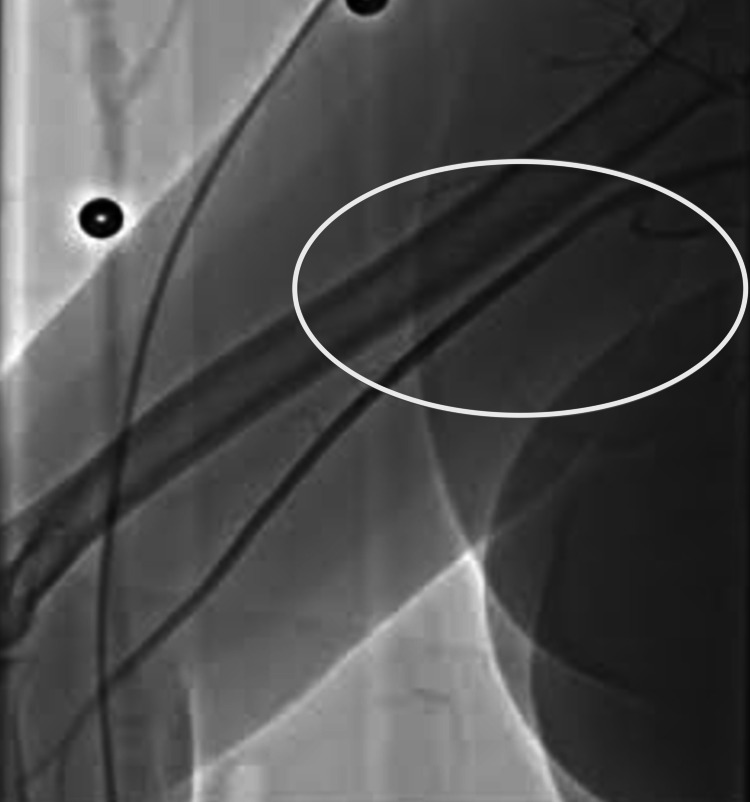
Right axillary artery follow-up

**Figure 6:**
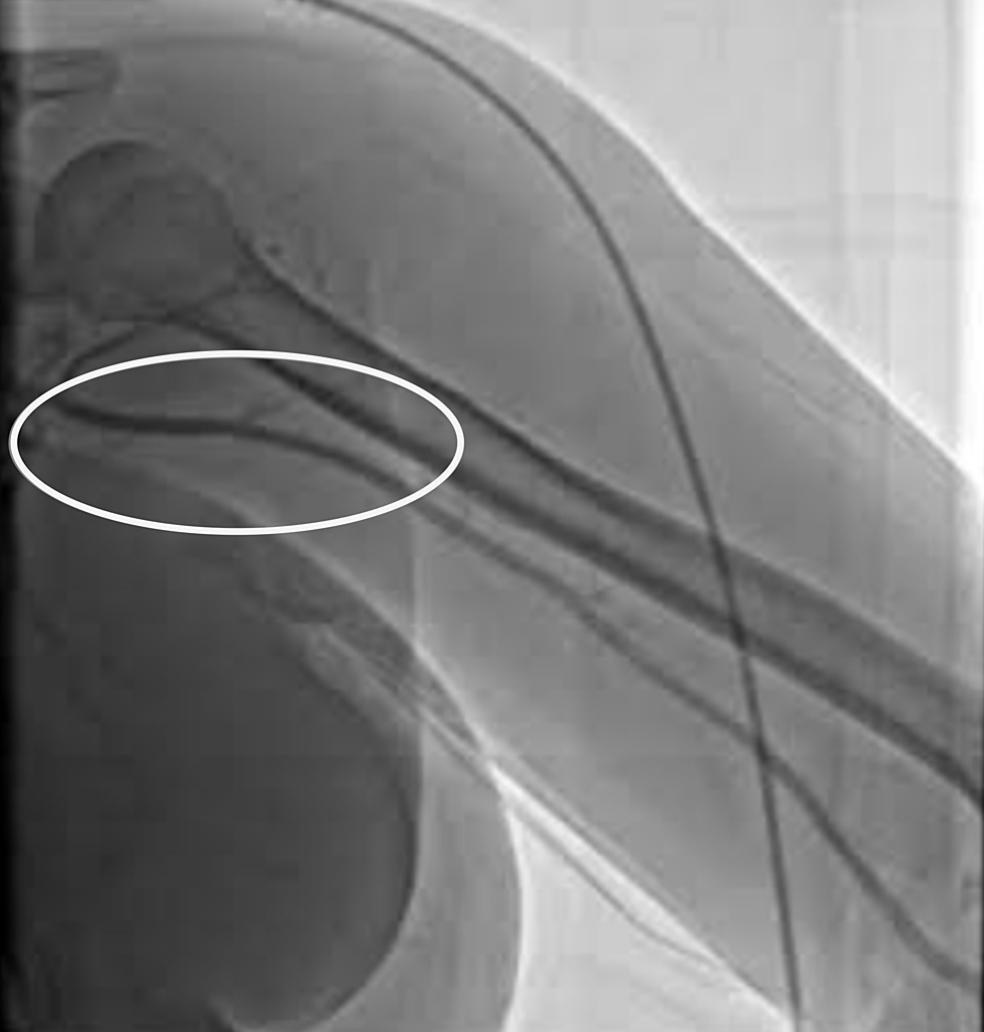
Left axillary artery follow-up

The patient’s results from the NV duplex ultrasound of the upper extremity arteries in November 2019 were as follows: Right upper extremity triphasic waveforms were seen in the right innominate artery and throughout the right subclavian artery. Biphasic waveforms were seen in the right axillary artery and throughout the right brachial artery, right radial artery, and right ulnar artery. In the left upper extremity, triphasic waveforms were seen in the proximal subclavian artery, and biphasic waveforms were seen in the mid and distal subclavian artery. Triphasic waveforms were seen in the left axillary artery, and biphasic waveforms in the brachial artery. Figure 5 demonstrates continued patency of the right axillary artery at follow-up and Figure 6 demonstrates continued patency of the left axillary artery at follow-up.

## Discussion

Peripheral artery disease of the upper extremities is less common than the lower extremities, and stenosis of the axillary artery is a rare occurrence that may be because of collateral circulations that exist [1]. Thorough documentation of percutaneous angioplasty for the axillary artery does not exist, and studies specifically focused on axillary artery stenosis need to be performed for physicians to understand the most effective treatment modalities better.

In the case of upper extremity peripheral artery disease, the most prevalent locations involved include the subclavian arteries [1]. Typically, asymptomatic patients can be medically managed, but revascularization is indicated if the patient is experiencing severe, disabling symptoms or if the patient has bilateral stenosis [1]. Clinical manifestations of upper extremity peripheral artery disease can include acute ischemic pain with exertion, chronic ischemic pain, and episodes of dizziness or syncope from potential retrograde flow due to severe stenosis [2].

A considerable drawback to the use of percutaneous transluminal angioplasty is a restenosis rate ranging from 40% to 60% at one year [3]. Restenosis is often seen as a result of neointimal hyperplasia. The solution to this is the use of drug-eluting devices, which include stents and balloons. The drug typically used to inhibit neointimal growth is paclitaxel. Complications, including stent thrombosis or bleeding from the required use of dual antiplatelet therapy, limit the overall benefit of stent placement. However, drug-eluting balloon angioplasty may circumvent this shortcoming [3]. According to a study done regarding paclitaxel-coated balloons' effectiveness, the balloons lost approximately 6% of the paclitaxel dose, and approximately 80% of the drug was released during inflation of the balloon [3]. This dosage released from the balloon may be an adequate percentage based on this patient’s results. There is currently no study in which a drug-eluting balloon has been used for restenosis in the upper arteries. A few case reports on use in subclavian and dialysis fistula restenosis are available.

Currently, there is no clear guidance for the treatment of axillary artery stenosis as it is quite rare. Current treatment of axillary artery stenosis is guided by arterial angiography combined with the patient’s symptomatology. The method of revascularization to be performed is at the discretion of the specialist.

The 2017 European Society of Cardiology (ESC) recommendations for the treatment of peripheral artery disease of the upper extremities state that surgical revascularization or stenting be used to treat symptomatic patients with subclavian artery stenosis [1]. Based on one study, balloon-coated angioplasty may provide an effective revascularization method without the need for a sustained drug-releasing option or the need for stenting [4].

## Conclusions

Here we present a case of bilateral peripheral artery disease of the upper extremities and, specifically, stenosis of the axillary arteries with restenosis. Stenosis of the axillary arteries was visualized by angiography and ultrasonography. The patient underwent revascularization of the axillary arteries along with the subclavian and brachial arteries. She did require repeat revascularization of the left subclavian, axillary, and brachial arteries one year later using a drug-eluting balloon, but she has since been asymptomatic and has had adequate flow based on ultrasound results. As there are no formal guidelines currently in place, we suggest percutaneous transluminal angioplasty as a successful regimen for the treatment of axillary artery stenosis. We would also suggest considering the use of a primary drug-eluting balloon to improve primary patency.
